# A rare case of bilateral elastofibroma dorsi of the back with postoperative subthoracic seroma: Case report

**DOI:** 10.1097/MD.0000000000042619

**Published:** 2025-07-11

**Authors:** Lin-Cen Xie, Shun-Bin Shi, Hai-Long Wang, Xiang You

**Affiliations:** a Department of Thoracic Surgery, Suzhou Ninth People’s Hospital, Suzhou, China.

**Keywords:** elastofibroma dorsi, postoperative seroma, surgery

## Abstract

**Rationale:**

Elastofibroma dorsi is a slow-growing, ill-defined benign soft tissue tumor characterized by excessive proliferation of fibroblast tissue. Tumors are made up of collagen and coarse elastic fibers. At present, the pathogenesis is unknown, and the incidence is extremely low.

**Patient concerns:**

A middle-aged and elderly male was admitted to our hospital, with chronic chest pain and palpable bilateral symmetrical dorsal subscapular mass.

**Diagnoses:**

Computed tomography showed the presence of bilateral soft tissue tumors, considering bilateral elastofibroma dorsi.

**Interventions and outcomes:**

The lesion was completely removed by surgery.

**Lessons:**

Elastofibroma dorsi is a relatively rare benign soft tissue tumor of the chest wall. For symptomatic patients with large tumor volumes, local tumor resection is feasible. It is recommended to routinely place negative pressure drainage tubes during surgery to effectively prevent postoperative local fluid accumulation. For postoperative patients with concurrent local fluid accumulation, the treatment effect of re-catheterization and drainage is good.

## 1. Introduction

Elastofibroma dorsi (ED) is a slow-growing, ill-defined benign soft tissue at present, its pathogenesis is unknown and its incidence rate is extremely low. Recently, our hospital admitted a middle-aged and elderly male with bilateral elastofibroma dorsi. The following is a report to strengthen our understanding of the disease and reduce unnecessary biopsies and surgeries.

## 2. Case presentation

**Figure 1.**
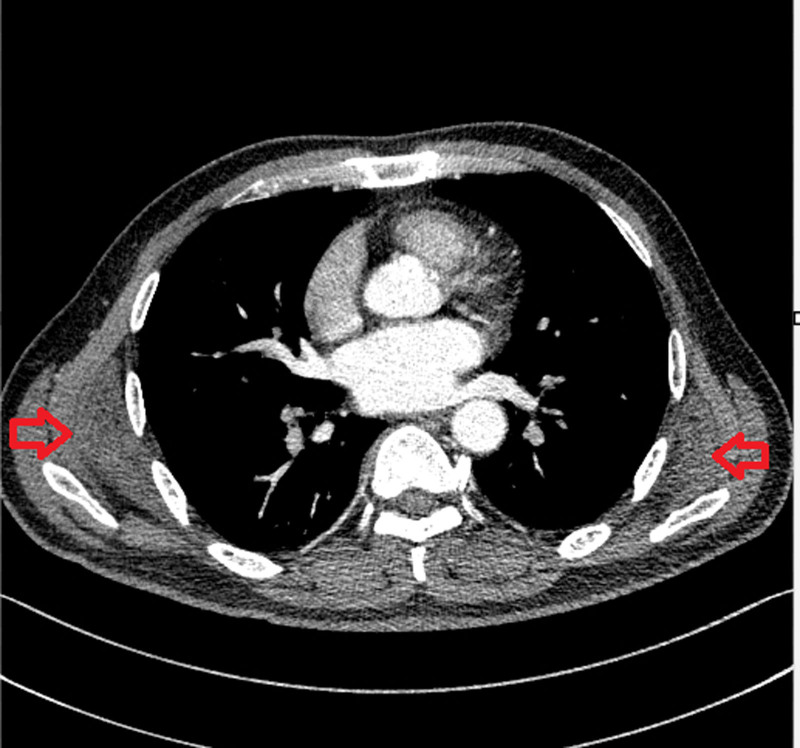
The preoperative CT showed that the density of the masses below the scapula, posterior medial to the serratus anterior muscle, and anterior latissimus dorsi on both sides was slightly low-density, and the density was uniform, similar to the surrounding muscle tissue, and the CT value was about 40 Hu, with blurred boundaries and symmetry. Enhancement scan enhancement is not obvious.

A patient, a male, 57-years old, experienced pain in both sides of the back for 2 years and gradually worsened for 1 month. In April 2024, a lump was discovered on both sides of the back, and sought treatment at the Ninth People’s Hospital of Suzhou. He complained of bouncing sounds during shoulder joint movements on both sides. Upon admission, a lump of approximately 8 cm × 4 cm and 11 cm × 5 cm could be palpated at the subscapular angle on both sides of the back. The lump was hard, fixed, painless, and had no fever or swelling on the surface of the skin. The limb and joint movements were unobstructed. When the anterior curvature of the shoulder was abducted, a circular lump could be seen sliding out of the subscapular angle. The laboratory examination showed no abnormalities. Perform computed tomography (CT) examination in our hospital, as shown in Figure 1.

### 2.1. Preoperative imaging diagnosis

**Figure 2.**
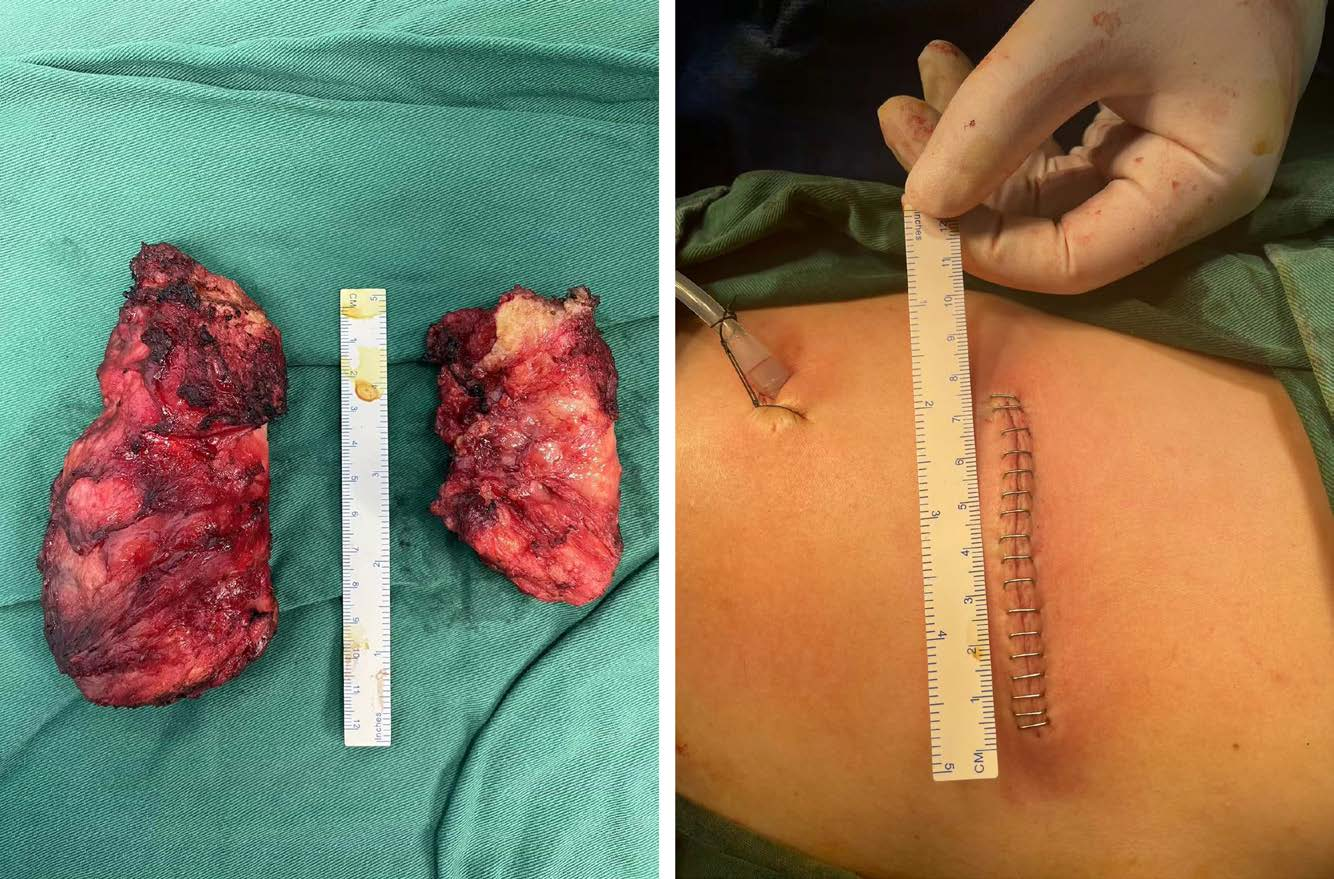
Elastofibroma has no intact capsule, but no obvious external invasion, similar to local neoplastic changes, tough, grayish-white in color. The surgical incision size is about 7 cm.

Imaging diagnosis: there may be elastic fibroids on both sides of the chest wall, please combine with clinical practice. Preoperative diagnosis considers the possibility of elastic fibroids on both sides of the chest wall, and malignancy cannot be ruled out. After informing the patient and their family of the relevant condition, routine pathology can be performed directly after surgery. Surgical method: on April 16th, the patient underwent bilateral back mass resection under general anesthesia by alternating positions. Take a horizontal incision of about 7 cm along the dermatoglyphic direction at the inferior angle of the affected side of the scapula, cut it to the muscular layer of the back, blunt separation, and locate the tumor. The superficial surface of the tumor is located on the deep surface of the back muscle at the lower corner of the scapula, and the deep surface of the tumor is closely attached to the surface of the rib. The boundary between the superficial and deep surfaces of the tumor is clear, with membranous adhesions. The surrounding muscles and fascia are tightly adhered to the chest wall. The boundary between the tumor and surrounding tissues is unclear, with no complete capsule, but no obvious external invasion, similar to local tumor-like proliferation changes, tough texture, and gray-white color. Remove the tumor and a small amount of surrounding connective tissue. The left mass is approximately 8 cm × 4.5 cm × 3 cm in size, and the right mass is approximately 11 cm × 5.5 cm × 5 cm in size, as shown in Figure 2. Negative pressure drainage was placed in bilateral surgical fields.

### 2.2. Postoperative pathology diagnosis

**Figure 3.**
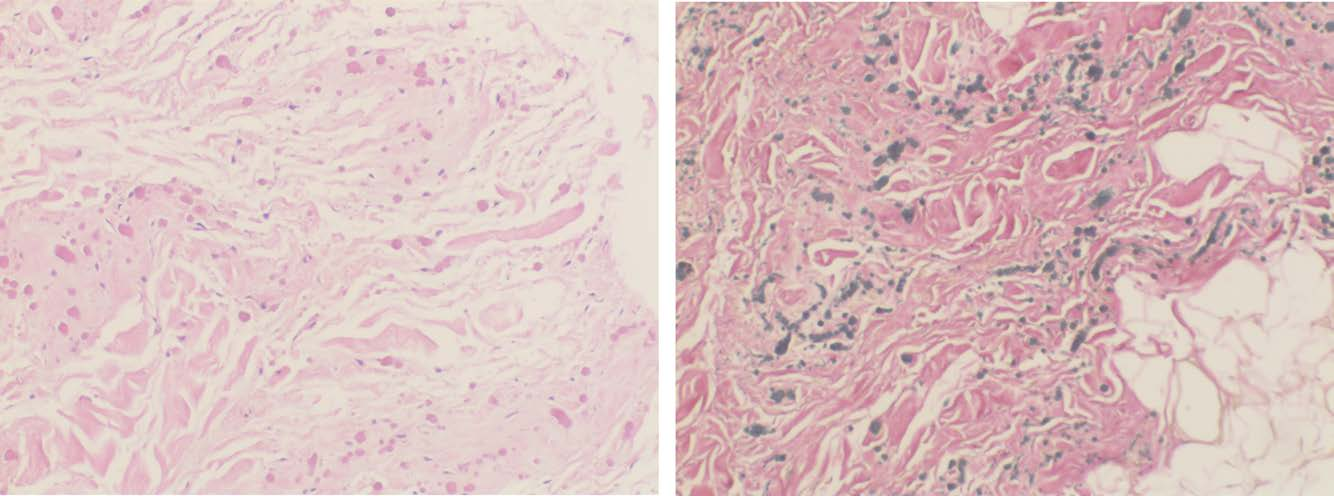
Under the microscope, elastic fibers of varying thicknesses are arranged in parallel and appear light red in color. The matrix is partially glassy degeneration of collagen fibers, and mild-shaped fibroblasts can be seen, as shown in the lesion contains scattered mature adipose tissue and vascular components, with unclear boundaries.

Pathologic diagnosis: under the microscope, elastic fibers of varying thicknesses are arranged in parallel and appear light red in color. The matrix is partially glassy degeneration of collagen fibers, and mild-shaped fibroblasts can be seen. The lesion contains scattered mature adipose tissue and vascular components, with unclear boundaries as shown in Figure 3.

### 2.3. Postoperative treatment

**Figure 4.**
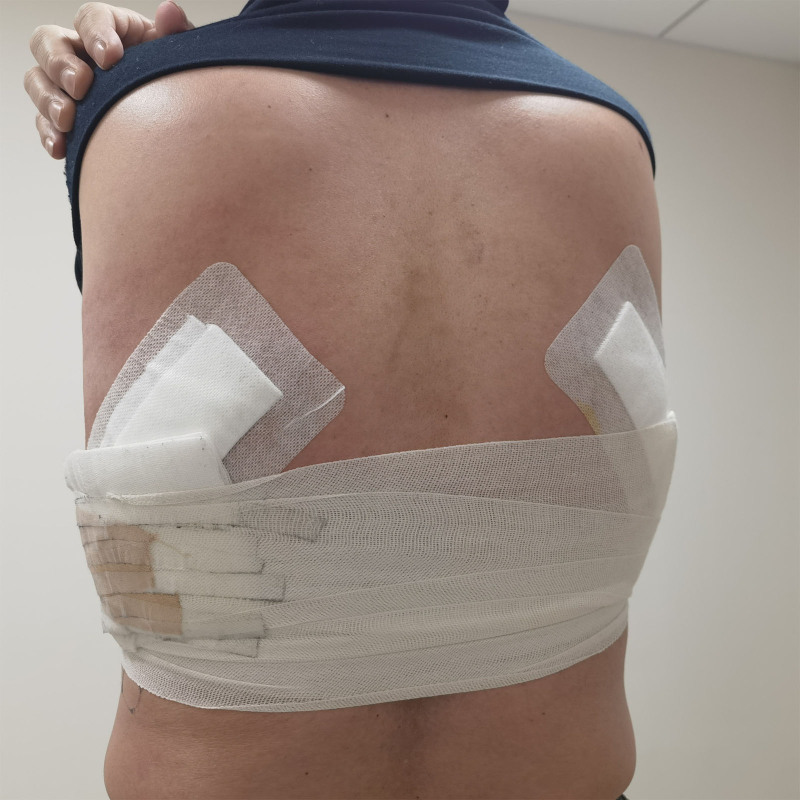
Continue bilateral elastic bandage chest fixation after getting out hospital.

After surgery, if the bilateral chest wall drainage is <30 mL, the chest wall drainage tube should be removed and discharged. After discharge, instruct him to continue to use elastic bandages to fix the chest as shown in Figure 4.

### 2.4. Postoperative subthoracic seroma and treatment

**Figure 5.**
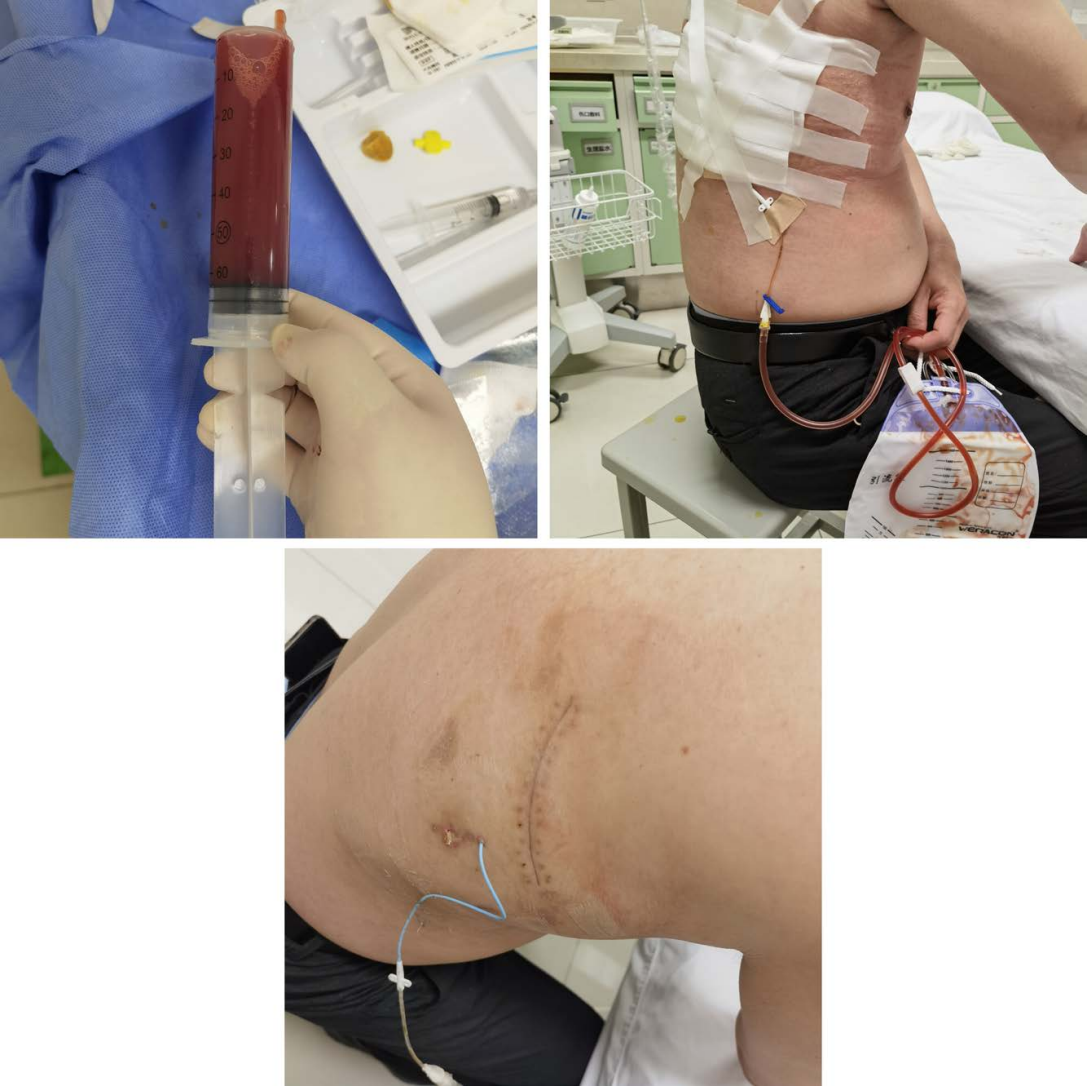
Bilateral chest wall catheterization drainage reveals pale bloody fluid. After 2 weeks of drainage, the chest wall hematoma disappeared.

One week later, a follow-up ultrasound revealed subthoracic effusion. Bilateral chest wall catheterization was performed to drain the fluid, revealing mild hematogenous fluid and bilateral seromas. The drainage was continued for 2 more weeks, with a drainage volume of <10 mL per day. No pleural effusion was detected during the follow-up ultrasound, and the fluid was removed as shown in Figure 5.

### 2.5. Recovery

**Figure 6.**
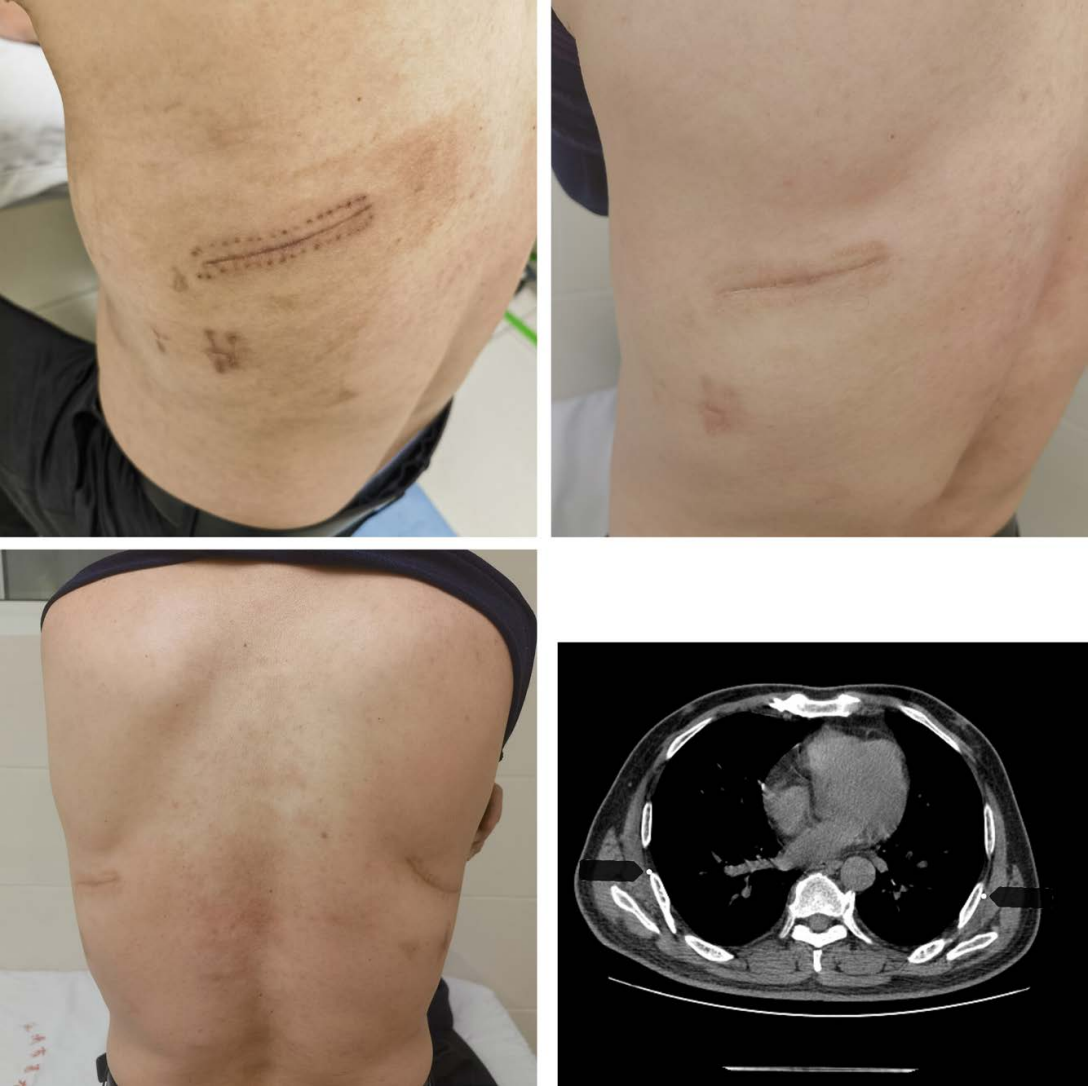
After 1 month and 11 months of follow-up, the incision healed well and with no recurrence after reexamination.

After 1 month and 11 months of follow-up, the general condition was good with no recurrence, and the wound healing was good as shown in Figure 6.

## 3. Discussion

Elastic fibroadenoma of the back is an extremely rare lesion, which was first reported by Jarvi and Saxen in 1961. Elastic fibroids are more common in middle-aged and elderly people and are more common in older women.^[1]^ Jarvi et al^[2]^ that in 235 autopsies, the detection rate of elastic fibroids in the back was as high as 17%, with women having a detection rate about twice that of men.

The pathogenesis of back fibroelastoma is still unclear, and there is still debate over whether it is a true tumor or a reactive pseudotumor. Some scholars believe that chronic friction and strain between the chest and scapula can cause local fibroelastosis and collagen degeneration,^[3]^ which is a pathogenic factor of back fibroelastoma and a response of local tissue to injury. It is a degenerative disease of fibrous connective tissue and a nontrue tumor rich in elastic fibrous tissue. This disease is more common in rural manual workers. The patient mentioned in this article is also. Thirty-two percent of patients with elastic fibroids reported by Nagamine et al^[4]^ have a family history. This disease needs to be differentiated from lipomas, sarcomas, desmoid tumors, fibromuscular tumors, soft tissue tumors, etc.^[3,5]^

Elastic fibroma of the back is most common in the area below the scapula, connected to the angle below the scapula, and often occurs on both sides. However, bilateral lesions can occur simultaneously or not simultaneously, with a few occurring in ischial nodules or soft tissues adjacent to the greater trochanter of the femur.^[6]^ The mass is often indistinct, hard, and tough, and most patients have no obvious symptoms. It is often discovered by chance, and rare symptoms include pain and discomfort below the scapula, making noises when the scapula moves, or limited shoulder movement.^[7]^ After resection, it does not recur. The typical imaging manifestation^[8]^ is symmetrical lesions below the scapulae on both sides. CT shows slightly lower or equal density, MRI shows equal T1 and slightly longer T2 signal shadows, and contrast-enhanced scans show moderate or significant enhancement. The lesions are more clearly displayed. Considering the small number of cells in the histology of elastic fibroids, which are nonspecific fibrous and adipose tissues. The diagnostic value of puncture is limited, and it is recommended to treat circular masses that slide out of the subscapular angle during adduction of the anterior curvature of the shoulder. Cases with typical imaging manifestations do not require preoperative puncture histological examination. Retrospective analysis showed that this patient presented with typical clinical and imaging features of elastic fibroadenoma in the back. Due to sufficient interdisciplinary discussions and literature review before surgery, the characteristics of this disease were recognized, and puncture was avoided in asymptomatic patients.

Due to the lack of reported malignant lesions in elastic fibroids and the fact that they do not affect upper limb movement, there is currently no recognized surgical indication standard. However, for patients with limited functional activity, compression symptoms, pain, or significant (>5 cm) lesions, complete surgical resection and marginal resection are recommended.^[9,10]^ The elastic fibroma in the back is tightly adhered to the surrounding chest wall muscles and fascia. Unclear boundaries can be achieved by cutting along the edge of the tumor to achieve an extremely low recurrence rate. This patient underwent resection along the edge of the tumor, and no recurrence was observed after surgery. The incidence of postoperative local incision effusion is relatively high.^[11]^ This patient developed local fluid accumulation at the incision after surgery and was cured after the placement of a drainage tube. Therefore, attention should be paid to stable hemostasis during surgery, and negative pressure drainage tubes should be placed routinely after surgery. For patients with postoperative local fluid accumulation, the treatment effect of placing tubes for drainage and to use elastic bandages to fix the chest is good.

In summary,^[12–15]^ elastic fibroadenoma of the back is a relatively rare benign soft tissue tumor of the chest wall. For patients with typical clinical manifestations and masses located in the subscapular or subscapular area with characteristic CT imaging features, the diagnosis is not a problem. For symptomatic patients with large tumor volumes, local tumor resection is feasible. It is recommended to routinely place negative pressure drainage tubes during surgery to effectively prevent postoperative local fluid accumulation. For postoperative patients with concurrent local fluid accumulation, the treatment effect of re-catheterization and drainage is good.

## Author contributions

**Funding acquisition:** Hai-Long Wang.

**Methodology:** Lin-Cen Xie.

**Supervision:** Shun-Bin Shi.

**Validation:** Xiang You.
